# Design of reference populations for genomic selection in crossbreeding programs

**DOI:** 10.1186/s12711-015-0104-x

**Published:** 2015-03-07

**Authors:** Ilse EM van Grevenhof, Julius HJ van der Werf

**Affiliations:** Animal Breeding and Genomics Centre, Wageningen University, Wageningen, The Netherlands; School of Environmental and Rural Science, University of New England, Armidale, Australia

## Abstract

**Background:**

In crossbreeding programs, genomic selection offers the opportunity to make efficient use of information on crossbred (CB) individuals in the selection of purebred (PB) candidates. In such programs, reference populations often contain genotyped PB animals, although the breeding objective is usually more focused on CB performance. The question is what would be the benefit of including a larger proportion of CB individuals in the reference population.

**Methods:**

In a deterministic simulation study, we evaluated the benefit of including various proportions of CB animals in a reference population for genomic selection of PB animals in a crossbreeding program. We used a pig breeding scheme with selection for a moderately heritable trait and a size of 6000 for the reference population.

**Results:**

Applying genomic selection to improve the performance of CB individuals, with a genetic correlation between PB and CB performance (r_PC_) of 0.7, selection accuracy of PB candidates increased from 0.49 to 0.52 if the reference population consisted of PB individuals, it increased to 0.55 if the reference population consisted of the same number of CB individuals, and to 0.60 if the size of the CB reference population was twice that of the reference population for each PB line. The advantage of using CB rather than PB individuals increased linearly with the proportion of CB individuals in the reference population. This advantage disappeared quickly if r_PC_ was higher or if the breeding objective put some emphasis on PB performance. The benefit of adding CB individuals to an existing PB reference population was limited for high r_PC_.

**Conclusions:**

Using CB rather than PB individuals in a reference population for genomic selection can provide substantial advantages, but only when correlations between PB and CB performances are not high and PB performance is not part of the breeding objective.

## Background

Traits expressed in purebred (PB) and crossbred (CB) individuals are genetically not the same [1]. PB and CB performances can be considered as two genetically correlated traits; the correlation between PB and CB performances (r_PC_) is affected by the extent of non-additive genetic effects (particularly dominance) and the genetic distance between lines or breeds crossed. This correlation also often reflects genotype by environment interactions due to nucleus PB and commercial CB animals being exposed to different environments. Wei and van der Werf [2] proposed to consider PB and CB performances as two correlated traits and to use a multi-trait model for their genetic evaluation. The breeding objective usually focuses on the performance of CB animals.

In practice, it is often difficult to use CB information since both performance and pedigree records can be difficult to obtain on CB animals. Moreover, selection for CB performance is either on half-sibs, which does not exploit variation within the half-sib family, or on progeny information, which would lengthen the generation interval if it was used. Bijma and van Arendonk [3] showed that extensive use of sib information on CB individuals can lead to increased rates of inbreeding.

Genomic selection could benefit crossbreeding programs since it allows using information on CB animals that is available at an early age and the method uses within-family variation [4,5]. Dekkers [6] proposed to use marker information that was calibrated based on the performance of commercial CB animals. He found a significant increase in the rates of genetic gain compared to using only PB phenotypic information, or combined PB and CB information, whereas the rate of inbreeding decreased.

Genomic selection uses marker genotypes and phenotypes in a reference population to predict breeding values of selection candidates that have been genotyped [7]. The effectiveness of genomic selection will depend on the size and composition of the reference population used for genomic predictions [8,9]. In crossbreeding programs, the breeding objective often includes CB performance, or in some cases, both PB and CB performance. Therefore, it seems reasonable to recommend the use of performance and genotypic data on CB individuals for genomic selection in crossbreeding programs. However, although large amounts of phenotypic as well as genotypic information on PB animals are usually already available, collecting CB information might be difficult, expensive and time-consuming. Thus, it is relevant to evaluate the benefit of using CB information in a reference population compared to that of PB information.

Dekkers [6] found that genetic gains were substantially less when genomic prediction was based on PB phenotypic data compared to CB phenotypic data. However, this assessment was based on comparisons for which the molecular breeding value (*mbv*) was combined with PB phenotypic information only, and the *mbv* was based on either PB or CB individuals with phenotypes, but not on both. Also, accuracies of *mbv* based on PB or CB individuals were assumed to be the same. However, if the linkage phase between a marker and a quantitative trait locus (QTL) is not consistent across breeds or selection lines, then a CB animal will have only one haplotype that is potentially informative to predict the *mbv* of a PB selection candidate whereas a PB animal has two relevant haplotypes. For a reference population of same size, phenotyping the CB individuals would require the estimation of twice the number of chromosome segments, therefore contributing less information per animal to a particular PB line. Nevertheless, the information provided is for the more relevant trait (CB performance).

This study aimed at assessing more generally the benefit of including CB information in the reference population of a crossbreeding program using genomic selection. The efficiency of investing in CB information to enable genomic selection of PB animals for CB performance was explored by varying the proportion of PB and CB animals in a reference population, or by adding CB individuals to an existing PB reference population. The size of the reference population, the size of the correlation between PB and CB performance and the emphasis on PB performance in the breeding objective were varied. Additionally, two examples of hard-to measure traits, relevant to pig breeding schemes, were considered.

## Methods

Selection index methodology provides a suitable framework to predict the accuracy of estimated breeding values for various breeding program scenarios, including the use of genomic information [10]. Deterministic simulation was used to predict the accuracy of estimated breeding values of selection candidates in a two-way crossing system. PB animals were selected for an index that included varying amounts of records and genotypes on CB and PB individuals. In scenarios with genomic selection, the make-up of the reference population was varied in terms of size, and proportion of CB individuals.

### Breeding program

We assumed a pig breeding nucleus with two PB lines that included 500 breeding females and 25 females mated to one male. We assumed that in each full-sib family, two males and two females were measured to become selection candidates for nucleus replacement. Nucleus replacements were selected on an index measured at a fixed time of selection.

For the base situation, we considered a trait that could be measured on both sexes before selection started. The trait heritability was 0.25. When selection started, phenotypic information was available on own performance, sire and dam performance, three full-sib records and 40 half-sib records of PB family members. To be able to compare the results of our study with those reported in [6], these parameters were set to the same values as in [6]. PB animals were mated to produce CB animals. The breeding goal was to improve the performance of CB animals, although, in some breeding schemes, PB performance also had some economic value. PB individuals had 10 CB half-sibs that were phenotypically measured at the time of their selection (no CB progeny). PB animals could also be genotyped before selection and the reliability of their genomically estimated breeding values depended on size and composition of the reference population. We assumed a reference population of varying size and with a varying proportion of CB animals.

We considered PB performance and CB performance as two genetically correlated traits, with correlation r_PC_. Parameters for the base scenario are described in Table 1.

**Table 1:** **Base parameters used in the simulations**

	**PB/CB**
Heritability, h^2^	0.25
Phenotypic standard deviation, SD	1
Common environment among full-sibs, c^2^	0.15
Economic value, PB_EV / CB_EV	0/1
Effective population size, N_e_	100
Reference population size, n_P_	6000
Purebred-Crossbred correlation, r_PC_	0.7

We also studied two single-trait examples relevant to pig breeding schemes that can be considered as ‘hard-to-measure traits’ and which therefore are expected to benefit more from genomic selection. We considered two sow traits that had different heritabilities and that could be measured within the breeding program. These traits were number of piglets born alive in first parity (PBA1) and length of productive life (LPL). PBA1 phenotypes were available for young sows and the heritability was 0.12 [11]. We evaluated scenarios based on accuracy of male selection. Males were selected based on information on the dam, two full-sib records, 20 half-sib records and five half-sib CB records. For genomic selection, we assumed that a total of 4000 records were available in the reference population of which 1000 were PB records and 3000 were individuals with a varying proportion of CB animals (p_CB_).

LPL is a measure of longevity that is only available on CB sows (because PB dams are kept only for a short time to limit generation interval) and late in life. Without genomic selection, this trait cannot be selected for in breeding programs. Genomic selection creates the potential to select on CB performance via genomic breeding values. LPL was assumed to have a heritability of 0.06 in CB individuals [11]. For genomic selection, we assumed a reference population of 6000 individuals, with p_CB_ ranging from 0 to 1. We assumed that, for the purpose of creating a reference population, some PB sows will be kept longer and measured for the trait, without actually being used as nucleus dams. The size of this reference population may seem unrealistically large, but such a size is required in order to obtain a reasonable genomic prediction accuracy for such a lowly heritable trait. For both traits, r_PC_ and EV_PB were varied.

### Accuracy of molecular breeding values

The accuracy *r*_*ag*_ of the estimated breeding values based on genomic information (*g*) was derived from the size and composition of total reference population. We considered two reference populations, one for PB and one for CB of size (1-p_CB_) · n_P_ and p_CB_ · n_P_, respectively, n_P_ is the size of the combined reference population. We used the formula of Daetwyler *et al*. [8] to predict *r*_*ag*_ of both *g*_*PB*_ and *g*_*CB*_: r_ag_ = h^2^/(h^2^ + λ), where h^2^ is the trait heritability and λ = n_g_/N where N is size of reference population (N =  (1-p_CB_) · n_P_ for purebreds and N = p_CB_ · n_P_ for crossbreds), n_g_ refers to the effective number of chromosome segments (independent loci). As suggested by Hayes *et al*. [12], n_g_ can be approximated as 2N_e_L, where N_e_ is the effective population size and L is the genome length in Morgans. For the pure line breeding population, we assumed an effective size of Ne = 100. We assumed that in the CB reference population, the number of chromosome segments to be estimated is twice that in the PB reference population, since, at each segment of the genome, the haplotypes originate from two different breeds or lines. Note that this is equal to assuming that each CB individual is half as informative as a PB individual (as 2n_g_/N = n_g_/ (N/2)).

### Selection index

Following Dekkers [6,10], multi-trait selection indices were derived to predict the accuracy of the selection index. We combined phenotypic information with genomic predictions of breeding value, i.e. the molecular breeding value (*g*), from both PB and CB information sources to estimate breeding values for PB (*a*_PB_) and CB (*a*_*CB*_) performances. For the selection index, the molecular breeding value *g* was considered as a separate trait, which is correlated to the breeding value it predicts, with the correlation between *a*_i_ and *g*_i_ (r_*aigi*_) equal to the accuracy of the molecular breeding value; the heritability of *g* was equal to 0.999. Hence, the multi-trait model used for the selection index contained four traits: *a*_PB_, *a*_CB_, *g*_PB_ and *g*_CB_. The correlation between *a*_PB_ and *a*_CB_ was equal to r_PC_ and the correlation between *g*_PB_ and *g*_CB_ was assumed to be equal to r_*a*PB*g*PB_.r_*a*CB*g*CB_.r_PC_, which are similar to those in [6] but we assumed that all the additive genetic variance was captured by markers, which in turn is an assumption consistent with Daetwyler *et al.* [8]. The selection index was optimized for the breeding objective with various degrees of emphasis on *a*_PB_ and *a*_CB_ and no emphasis on any of the *g*_i_. The accuracy of the selection index was used as a criterion to compare the performance of different scenarios.

We varied the size of the total reference population (n_P_), the degree of emphasis put on PB performance in the breeding objective (PB_EV), and the correlation between PB and CB performances (r_PC_). In all comparisons, we plotted the index accuracy versus p_CB_.

## Results

### Reference population structure

**Figure 1:**
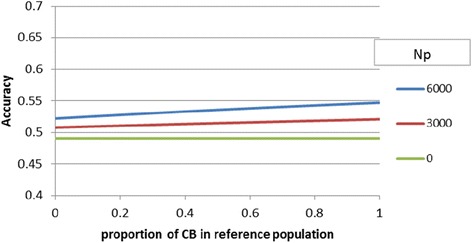
**Effect of the proportion of CB animals in the reference population on index accuracy of a breeding program using PB selection candidates selected on CB performance for various sizes of reference population.**

For the base scenario, where r_PC_ = 0.7 and the breeding objective aimed at improving CB performance, the index accuracy based on PB phenotypic information alone was 0.45. Combining phenotypic information on PB and CB animals gave an accuracy of 0.491, i.e. 10% higher than that obtained with PB information alone. Index accuracy increased to 0.526 if, in addition, genomic selection was based on a PB reference population of 6000 individuals. The best scenario used genomic selection based on 6000 CB individuals, giving an index accuracy of 0.554. In general, increasing the proportion of CB animals in the reference population increased the index accuracy (Figure 1), but this increase was relatively small in the base scenario. The relative increase in index accuracy due to genomic selection was 7.3% (0.491 vs. 0.526) if the reference population included PB animals and was 12.8% (0.491 vs 0.554) if a CB reference population of equal size was used. For a large reference population (n_p_ = 6000), the difference between using a PB reference population versus a CB reference population was a relative increase in accuracy of 5.2% (0.526 vs 0.554). The increase was nearly linear with the proportion of CB individuals in the reference population (p_CB_); a 50% CB/PB reference population resulted in about 2.8% greater accuracy than a pure PB reference population. The index accuracy also increased almost linearly with the size of the reference population (n_P_). For example, compared to no genomic selection, the index accuracy increased by 6.9% and 12.8% with a reference population of 3000 and 6000 CB individuals, respectively, and it increased by 3.9% and 7.3% with a reference population 3000 and 6000 PB, respectively.

### Breeding objective

**Figure 2:**
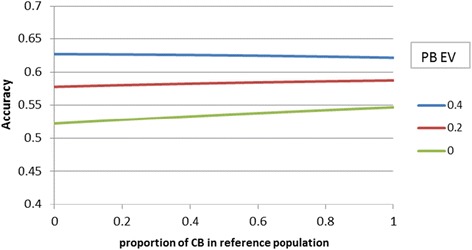
**Effect of the proportion of CB animals in the reference population on index accuracy of PB selection candidates when the emphasis put on PB performance varies in the breeding objective (PB_EV as fraction of CB_EV).**

Figure 2 compares the index accuracy for different breeding objectives and with the relative emphasis put on PB performance (PB_EV) ranging from 0 to 0.4. When PB performance had some economic value in the breeding objective (PB_EV > 0), then the benefit of having CB animals in the reference population decreased (Figure 2). The index accuracy increased by 5.2% when using a CB rather than a PB reference population if PB_EV = 0 but only by 1.8% (0.583 vs. 0.593) if PB_EV = 0.2, and there was a small loss of 1% in accuracy (0.633 vs. 0.626) if PB_EV = 0.4. This loss in accuracy is explained by the lower amount of information on genomic prediction accuracy delivered per CB animal since more haplotypes need to be estimated in a CB population. Accuracy of genomic prediction of *g*_*PB*_ from 6000 PB animals was predicted to be 0.46, whereas that of *g*_*CB*_ was only 0.33 when using 6000 CB animals.

### Correlation between PB and CB performances

**Figure 3:**
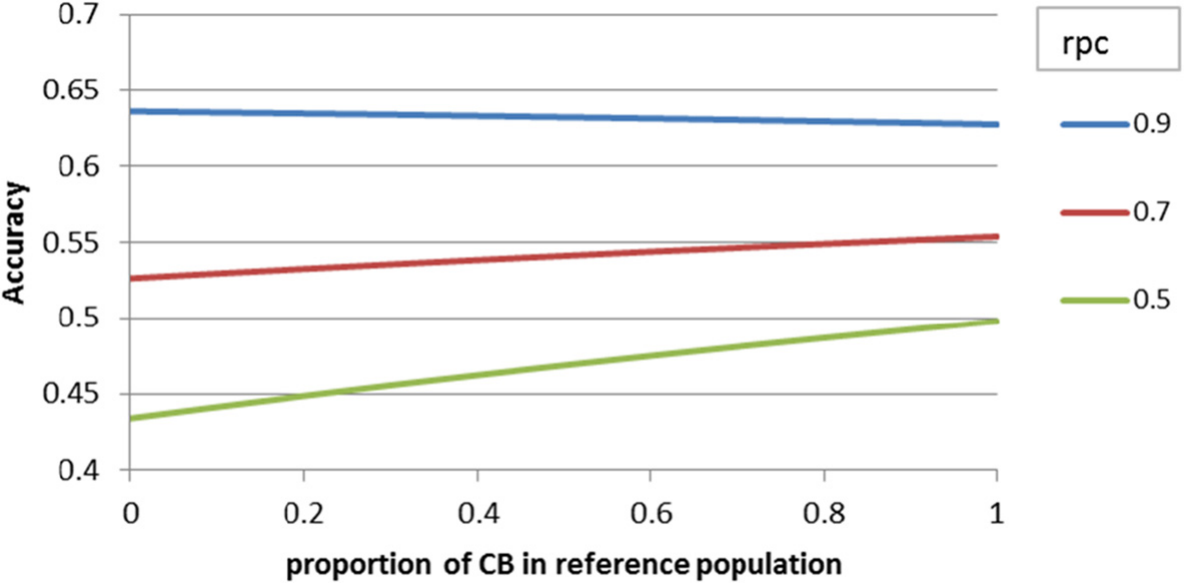
**Effect of the proportion of CB animals in the reference population on index accuracy of a breeding program using PB selection candidates selected on CB performance for various purebred-crossbred correlations (r**_**PC**_ **).**

Figure 3 shows the effect of r_PC_ on index accuracy and how the accuracy changes with p_CB_. For a high r_PC_ of 0.9, the effect of replacing PB by CB animals in the reference population was negative, again because of the lower prediction accuracy of *g*_*CB*_ vs. *g*_*PB*_. The index accuracy of genomic breeding programs using a PB reference population differed from a CB reference population with 5.2% for r_PC_ = 0.7 and 14.8% (0.434 vs. 0.498) for r_PC_ = 0.5. Again, the increase was nearly linear in p_CB_, so with p_CB_ = 0.5, this additional accuracy was about halved.

### Effect of CB individuals in a reference population

**Figure 4:**
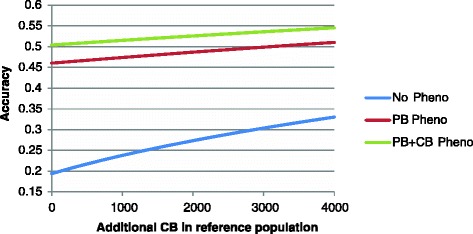
**Effect on index accuracy of a breeding program using PB selection candidates selected on CB performance when adding additional CB animals to a 2000 PB reference population, with or without phenotypic information available on selection candidates and their relatives.**

In practice, PB information is often available. Therefore, it would be interesting to estimate the benefit of adding CB individuals to an existing (potential) reference population when selecting for CB performance. Figure 4 shows the effect of adding CB animals to a PB reference population of 2000 individuals. The results show that index accuracy increased considerably if no phenotypic information was available on selection candidates, but this increase was relatively small if individual phenotypic measurements on selection candidates were available (either on PB only or on both PB and CB individuals). This illustrates that an increase in genomic prediction accuracy is less useful when more information is already available to estimate genetic merit. Although, the benefit of adding CB individuals to PB individuals in the reference population was small when phenotypes on the reference animals (either on PB only or on both PB and CB individuals) were available, the marginal benefit of adding CB animals to the reference population was higher than the benefit of adding PB individuals. For example, increases in index accuracy resulting from the addition of 2000 CB or 4000 PB to a reference population of 2000 PB individuals were the same. In the absence of phenotypic data on selection candidates, increases in index accuracy from adding 2000 CB or 2325 PB individuals to a reference population of 2000 PB are about the same, thus the benefit of adding CB over PB individuals was smaller for that scenario.

### Hard-to-measure traits

**Figure 5:**
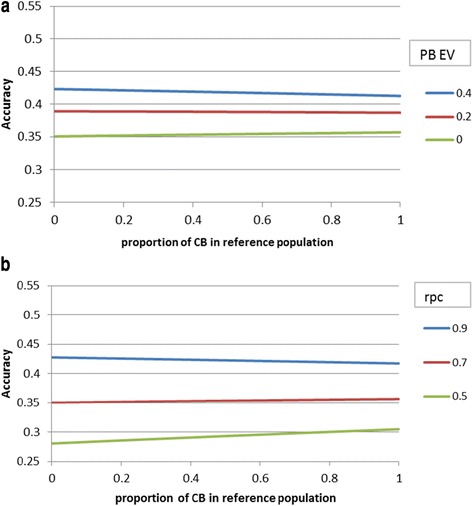
**Effect of the proportion of CB animals in the reference population (in addition to 1000 PB) on index accuracy of PB males for piglets born alive in first parity (PBA1) when varying the emphasis put on PB performance in the breeding objective (PB_EV as fraction of CB_EV) (a) or when varying purebred-crossbred correlations (r**_**PC**_ **) (b).**

**Figure 6:**
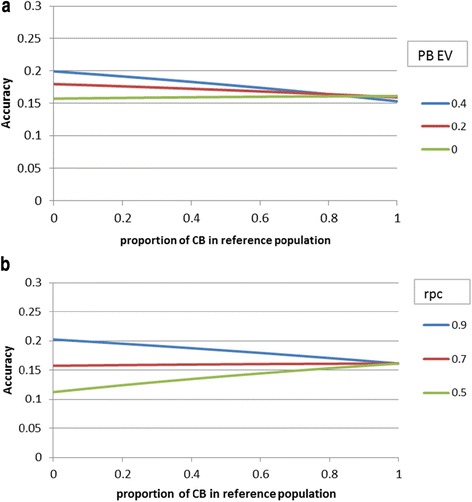
**Effect of the proportion of CB animals in the reference population on index accuracy of PB males for length of productive life (LPL) when varying the emphasis put on PB performance in the breeding objective (PB_EV as fraction of CB_EV) (a) or when varying purebred-crossbred correlations** **(r**_**PC**_ **) (b).**

Results for PBA1 are in Figures 5a and b and those for LPL are in Figures 6a and b. Results for these hard-to-measure traits confirm the earlier conclusion that the value of CB over PB individuals in the reference population depends highly on the r_PC_. Compared with the base scenario, the value of CB individuals in the reference population was slightly smaller compared to that of PB individuals in the reference population for the trait PBA1 and significantly smaller for the trait LPL. For both traits, there was no added value of CB over PB individuals in the reference population, unless the r_PC_ value was less than 0.7 (Figures 5b and 6b). Although less phenotypic information was available for PBA1 and LPL than for the base trait, the added accuracy obtained from the genomic information did not increase much compared to the base situation because either the size of the reference population was also smaller due to the sex-limited character of the trait (PBA1) or heritability of the trait was low (LPL). Because no phenotypic data on LPL was available for PB selection candidates, accuracies were the same for all r_PC_ values when reference populations consisted of only CB animals (Figure 6a). Figures 5a and 6a compare accuracies for breeding objectives with varying relative emphasis on PB performance (PB_EV). Since there was less phenotypic information on PB animals for LPL than for PBA1, the benefit of having PB information in the reference population was relatively greater for LPL (Figures 5a and 6a). This result is consistent with that of Figure 4, which shows that the benefit of replacing PB with CB individuals decreased when there was less phenotypic information on selection candidates.

## Discussion

Genomic selection can be very valuable in crossbreeding programs since it allows efficient selection on CB performance. In general, a larger proportion of CB animals in the reference population will increase the selection accuracy when the breeding goal is focussed on CB performance. However, it might be difficult and expensive to collect phenotypes and genotypes on CB individuals, whereas most breeding programs have routine phenotyping measurement and genotyping of nucleus animals in the pure lines. We found that the effect of replacing PB with CB animals in the reference population was highly positive but only when the correlation between PB and CB performance was low (r_PC_ < 0.7) and the breeding objective emphasis was mainly focused on improving CB performance. In our example, with an r_PC_ of 0.7 and a breeding objective that focused only on CB performance, the index accuracy increased from 0.526 to 0.554 when using a CB instead of a PB reference population. With an r_PC_ of 0.9, the index accuracy decreased slightly from 0.635 to 0.626 for a CB compared to a PB reference population. Therefore, the additional gain from using CB rather than PB individuals in the reference population decreased rapidly with higher values of r_PC_ and also when emphasis was put on PB performance in the breeding objective. The increase in index accuracy from including genomic information was also relatively low when selection of PB animals was already based on phenotypic information from selection candidates and their relatives (including CB).

Dekkers [6] concluded that marker-based selection in crossbreeding programs was much more effective when molecular prediction was based on commercial CB data rather than on PB data, which contradicts our findings that the additional gain from using CB animals in the reference set is in many cases only modest. Dekkers [6] used an r_PC_ of 0.7 and a breeding objective that targeted CB performance only. One difference between our study and that of Dekkers [6] is that Dekkers assumed that accuracies of *g*_*PB*_ and *g*_*CB*_ were equal whereas we assumed an equal number of phenotypes, which led to a lower prediction accuracy of *g*_*CB*_ versus *g*_*PB*._ If we assumed accuracies of *g*_*PB*_ and *g*_*CB*_ were equal, the index accuracy increased from 0.49 to 0.60 (23% increase) with a CB versus a PB reference population, instead of an increase from 0.49 to 0.55 (13% increase; Figure 1). One could argue that in practice a breeder would be interested in improving both selection lines, in which case the choice is between investing in genotyping n PB individuals in each line, or 2n CB individuals, the latter providing information for both lines. In that case, the molecular breeding values *g*_*PB*_ and *g*_*CB*_ would be equally accurate, and the benefit of investing the same genotyping effort in CB rather than PB animals would be higher than shown here. However, we also showed that knowledge of the r_PC_ is critical in assessing this benefit.

Another difference is that Dekkers [6] assumed accuracies of 0.6 for predictions of *g*_*PB*_ and *g*_*CB*_, which is higher than what we used here. Higher genomic prediction accuracy gives more gain in accuracy when replacing PB by CB animals in the reference population, as shown in Figure 1. Furthermore, Dekkers [6] compared the gains from using molecular information only, or adding molecular information to PB phenotypic information on the selection candidates. We assumed that some phenotypic information was known on 10 CB half-sibs, which makes the relative value of adding molecular information based on CB phenotypes smaller. If the phenotypic information on 10 CB half-sibs was discarded, the increase in index accuracy due to introducing genomic selection was 17% (from 0.445 to 0.520) if it was based on a CB reference population) but only 9% (from 0.445 to 0.486) if it was based on a PB reference population. If CB performance is measured, then there is some advantage in genotyping the same individuals, but similarly, if CB performance is measured for the reference population, then this information should be used to predict the breeding value of PB relatives. Genomic information can be used to predict relatedness, which alleviates the need for pedigree recording in the CB animals. Hence, genomic testing and phenotyping of CB individuals might provide more information than predicted by the Daetwyler *et al.* [8] formula (which assumes prediction from unrelated individuals), since it facilitates the use of information on close CB relatives.

We used a selection index approach to predict the additional gains from genomic information in breeding programs. The selection index model combines information from phenotypic data on relatives with genomic prediction, and assumes that these sources of information are independent. In practice, this is often not the case, e.g. CB animals can contribute to a reference population, but they can also directly affect predictions of relatives through pedigree relationships. A dependency between these sources of information needs to be accounted for in genetic evaluation procedures, e.g. in *ad-hoc* “blending” methods [13], or more appropriately in the so-called “single-step” method [14], to avoid bias and inflation of the accuracy of the combined breeding value. Such dependence may exist for some individuals, but not for others, so it is difficult to account for it in a deterministic modelling study. Moreover, ignoring this dependence may inflate genomic prediction accuracy, and therefore the benefit of genomic information, but it is unlikely to affect the relative value of CB vs PB records in the reference population.

In practice, breeding objectives often put emphasis on PB performance, in which case the added value of adding CB animals in the reference population becomes very small. The benefit of increasing the percentage of CB individuals in the reference population depends on r_PC_, as well as on PB_EV. In our examples, we found that if r_PC_ is not very low (~0.8) and PB_EV is greater than 0, which is a realistic scenario, then the benefits of using PB or CB animals in the reference population are similar.

In this study, we assumed an effective population size of 100. With Ne = 500, the added value of genomic selection based on a PB reference population was only 1.7% and increased to 2.9% if the reference population consisted of only CB animals. The accuracy of genomic prediction of *g*_PB_ based on 6000 PB animals was predicted to be 0.22 for Ne = 500, whereas that of *g*_CB_ was only 0.16 based on 6000 CB animals. Both predictions assume no direct pedigree relationships between the reference population and selection candidates and probably underestimate the accuracy achieved in practice.

The effective population size affects the estimated number of effective chromosome segments or independent loci (n_g_), and this can have a large effect on the predicted accuracy of molecular breeding values (r_*a,g*_). However, estimating n_g_ from N_e_ is not straightforward and approximations used in the literature differ [9,15-17]. We used n_g_ = 2N_e_L, following Hayes *et al*. [12]. Other approximations were proposed, e.g. Goddard [9] suggested n_g_ = 2NeL/log(4NeL), Goddard *et al.* [15] used n_g_ = 2NeLk/log(2Ne.L) and Meuwissen *et al.* [16] proposed n_g_ = 2NeLk/ln(2Ne), where L is the number of chromosomes and k is the average length per chromosome (i.e. their Lk is the same as our L). The latter approximation leads to a considerably smaller estimate for n_g_ and therefore higher accuracies. It might be useful to use empirical evidence to verify these predictions. However, the approximation of r_*a,g*_ has an impact on the benefit of genomic selection in breeding programs by affecting the size of the reference population required to achieve a certain benefit, but we showed that it has only a small impact on the optimal composition in terms of the proportion of CB phenotypes in the reference population.

This study did not take the Bulmer effect in consideration, which would reduce the gain due to the phenotypic information on CB half-sibs [17]. Hence, the benefit of genotyping CB animals rather than just using their phenotypes will be higher in reality than presented in this paper. Also, the effect on inbreeding will be favourable when applying genomic selection in crossbreeding programs. These advantages have been pointed out by Dekkers [6]. The main focus of this paper was to look at the effect of different proportions of PB and CB animals in the reference population, when applying genomic selection in CB programs, and these would not be much affected by the Bulmer effect.

Based on deterministic simulations, this paper shows the potential benefit of including CB information in crossbreeding programs, using pig breeding as an example. The main aim of the paper was to evaluate this benefit in relation to some key parameters. The actual value for these parameters, and therefore the value of using CB information will be case dependent. Some recent papers [18,19] discussed the use of genomic selection in pig breeding programs and the opportunities and challenges it brings along.

## Conclusions

Genomic selection for CB performance can significantly increase rates of genetic gain in crossbreeding programs. The rate of genetic gain increases more when CB animals are included in the reference population compared to a PB reference population. However, we found that the benefit of replacing PB animals with CB animals in the reference population is small, unless the correlation between PB and CB performance is lower than ~0.8 and PB performance is not considered in the breeding objective.
